# Supplementary material for: Mapping resilience: Development of the resilience process scales (RPS) and resilience profiles during adversity
Source: PLoS One. 2026 Feb 11;21(2):e0341581. doi: 10.1371/journal.pone.0341581 (PMC12893550; doi:10.1371/journal.pone.0341581)
Supplement: S1 Appendix — 20-item scale proposed during item development and examined in Study 1. (PDF) [file pone.0341581.s001.pdf]

Item number	Item	Process
1	I reflect and learn from difficult experiences.	Mend
2	I remain positive, even when things seem hopeless.	Manage
3	I make back-up plans for when things might go wrong.	Minimize
4	I tend to organise myself well to deal with challenges.	Minimize
5	I bounce-back easily after a challenge.	Mend
6	When things get bad, I don't let them get to me.	Manage
7	I can anticipate when help is going to be needed.	Anticipate
8	I quickly get over set-backs.	Mend
9	I keep a clear head under pressure.	Manage
10	I give my best effort no matter the obstacle.	Manage
11	I know how to stop the same things getting to me in the future.	Mend
12	I recognise when tough challenges are approaching.	Anticipate
13	I know when to get help after a tough situation.	Mend
14	I seek out support before stress affects me.	Minimize
15	I can anticipate when a situation will stress me.	Anticipate
16	I recover quickly after feeling worn-out.	Mend
17	I notice possible difficult situations early.	Anticipate
18	In stressful situations I usually maintain focus.	Manage
19	I prepare myself for upcoming challenges.	Minimize
20	I know where I can get help when difficult problems are approaching.	Minimize
