# Supplementary material for: Mapping resilience: Development of the resilience process scales (RPS) and resilience profiles during adversity
Source: PLoS One. 2026 Feb 11;21(2):e0341581. doi: 10.1371/journal.pone.0341581 (PMC12893550; doi:10.1371/journal.pone.0341581)
Supplement: S2 Appendix — 13-item Resilience Process Scales, with vignettes separating each domain, and the scoring system for the processes. (PDF) [file pone.0341581.s002.pdf]

**The Resilience Process Scales (RPS).**

**General**

Please think of different tough situations, life events, challenges and obstacles that you may have experienced in the past or may experience in the future. This could be any event in which you have, or could experience stress, pressure, or hardship.

Indicate below the extent to which you agree with each statement about yourself with regards to this/these experience(s), by circling the relevant number on the rating scale from "1" (Strongly Disagree) to "7" (Strongly Agree), with "4" being that you neither agree or disagree. Choose a number which best indicates your feelings about that statement. There are no right or wrong answers and all ratings will be kept confidential so please answer honestly.

Everyone differs in how they deal with different situations. It is likely that there will be areas that you are better or worse in; this is totally normal and something we would expect.

| In these situations… | Strongly Disagree | | | Neither agree or disagree | | | Strongly Agree |
|---|---|---|---|---|---|---|---|
| 1. I remain positive, even when things seem hopeless. | 1 | 2 | 3 | 4 | 5 | 6 | 7 |
| 2. I make back-up plans for when things might go wrong. | 1 | 2 | 3 | 4 | 5 | 6 | 7 |
| 3. I tend to organise myself well to deal with challenges. | 1 | 2 | 3 | 4 | 5 | 6 | 7 |
| 4. I bounce-back easily after a challenge. | 1 | 2 | 3 | 4 | 5 | 6 | 7 |
| 5. When things get bad, I don't let them get to me. | 1 | 2 | 3 | 4 | 5 | 6 | 7 |
| 6. I can anticipate when help is going to be needed. | 1 | 2 | 3 | 4 | 5 | 6 | 7 |
| 7. I keep a clear head under pressure. | 1 | 2 | 3 | 4 | 5 | 6 | 7 |
| 8. I quickly get over set-backs | 1 | 2 | 3 | 4 | 5 | 6 | 7 |
| 9. I give my best effort no matter the obstacle. | 1 | 2 | 3 | 4 | 5 | 6 | 7 |
| 10. I know how to stop the same things getting to me in the future. | 1 | 2 | 3 | 4 | 5 | 6 | 7 |
| 11. I can anticipate when a situation will stress me. | 1 | 2 | 3 | 4 | 5 | 6 | 7 |
| 12. I notice possible difficult situations early. | 1 | 2 | 3 | 4 | 5 | 6 | 7 |
| 13. I prepare myself for upcoming challenges. | 1 | 2 | 3 | 4 | 5 | 6 | 7 |

**The Resilience Process Scales (RPS).**

**Physical**

Please think of different tough **physical** situations, life events, challenges and obstacles that you may have experienced in the past or may experience in the future. This could range from exercise, sport, outdoors activities, illness, injury in which you have or may experience exhaustion, hunger, thirst, or any other physical issue.

In these situations…

| | Strongly Disagree | | | Neither agree or disagree | | | Strongly Agree |
|---|---|---|---|---|---|---|---|
| 1. I remain positive, even when things seem hopeless. | 1 | 2 | 3 | 4 | 5 | 6 | 7 |
| 2. I make back-up plans for when things might go wrong. | 1 | 2 | 3 | 4 | 5 | 6 | 7 |
| 3. I tend to organise myself well to deal with challenges. | 1 | 2 | 3 | 4 | 5 | 6 | 7 |
| 4. I bounce-back easily after a challenge. | 1 | 2 | 3 | 4 | 5 | 6 | 7 |
| 5. When things get bad, I don't let them get to me. | 1 | 2 | 3 | 4 | 5 | 6 | 7 |
| 6. I can anticipate when help is going to be needed. | 1 | 2 | 3 | 4 | 5 | 6 | 7 |
| 7. I keep a clear head under pressure. | 1 | 2 | 3 | 4 | 5 | 6 | 7 |
| 8. I quickly get over set-backs | 1 | 2 | 3 | 4 | 5 | 6 | 7 |
| 9. I give my best effort no matter the obstacle. | 1 | 2 | 3 | 4 | 5 | 6 | 7 |
| 10. I know how to stop the same things getting to me in the future. | 1 | 2 | 3 | 4 | 5 | 6 | 7 |
| 11. I can anticipate when a situation will stress me. | 1 | 2 | 3 | 4 | 5 | 6 | 7 |
| 12. I notice possible difficult situations early. | 1 | 2 | 3 | 4 | 5 | 6 | 7 |
| 13. I prepare myself for upcoming challenges. | 1 | 2 | 3 | 4 | 5 | 6 | 7 |

**The Resilience Process Scales (RPS).**

**Social**

Please think of different tough **social** situations, life events, challenges and obstacles you may have experienced in the past or may experience in the future. This could range from arguments, criticism you have had or may experience, public speaking, making new friends, and other difficult relationship, family and friendship issues.

In these situations…

| | Strongly Disagree | | | Neither agree or disagree | | | Strongly Agree |
|---|---|---|---|---|---|---|---|
| 1. I remain positive, even when things seem hopeless. | 1 | 2 | 3 | 4 | 5 | 6 | 7 |
| 2. I make back-up plans for when things might go wrong. | 1 | 2 | 3 | 4 | 5 | 6 | 7 |
| 3. I tend to organise myself well to deal with challenges. | 1 | 2 | 3 | 4 | 5 | 6 | 7 |
| 4. I bounce-back easily after a challenge. | 1 | 2 | 3 | 4 | 5 | 6 | 7 |
| 5. When things get bad, I don't let them get to me. | 1 | 2 | 3 | 4 | 5 | 6 | 7 |
| 6. I can anticipate when help is going to be needed. | 1 | 2 | 3 | 4 | 5 | 6 | 7 |
| 7. I keep a clear head under pressure. | 1 | 2 | 3 | 4 | 5 | 6 | 7 |
| 8. I quickly get over set-backs | 1 | 2 | 3 | 4 | 5 | 6 | 7 |
| 9. I give my best effort no matter the obstacle. | 1 | 2 | 3 | 4 | 5 | 6 | 7 |
| 10. I know how to stop the same things getting to me in the future. | 1 | 2 | 3 | 4 | 5 | 6 | 7 |
| 11. I can anticipate when a situation will stress me. | 1 | 2 | 3 | 4 | 5 | 6 | 7 |
| 12. I notice possible difficult situations early. | 1 | 2 | 3 | 4 | 5 | 6 | 7 |
| 13. I prepare myself for upcoming challenges. | 1 | 2 | 3 | 4 | 5 | 6 | 7 |

Classification: Controlled

**The Resilience Process Scales (RPS).**

**Mental**

Please think of different tough **mental** situations, life events, challenges and obstacles you may have experienced in the past or may experience in the future. This could range from exams, studies, puzzles, or other tough situations which required or may require concentration, decision making, and thinking skills.

| In these situations… | Strongly Disagree | | | Neither agree or disagree | | | Strongly Agree |
|---|---|---|---|---|---|---|---|
| 1. I remain positive, even when things seem hopeless. | 1 | 2 | 3 | 4 | 5 | 6 | 7 |
| 2. I make back-up plans for when things might go wrong. | 1 | 2 | 3 | 4 | 5 | 6 | 7 |
| 3. I tend to organise myself well to deal with challenges. | 1 | 2 | 3 | 4 | 5 | 6 | 7 |
| 4. I bounce-back easily after a challenge. | 1 | 2 | 3 | 4 | 5 | 6 | 7 |
| 5. When things get bad, I don't let them get to me. | 1 | 2 | 3 | 4 | 5 | 6 | 7 |
| 6. I can anticipate when help is going to be needed. | 1 | 2 | 3 | 4 | 5 | 6 | 7 |
| 7. I keep a clear head under pressure. | 1 | 2 | 3 | 4 | 5 | 6 | 7 |
| 8. I quickly get over set-backs | 1 | 2 | 3 | 4 | 5 | 6 | 7 |
| 9. I give my best effort no matter the obstacle. | 1 | 2 | 3 | 4 | 5 | 6 | 7 |
| 10. I know how to stop the same things getting to me in the future. | 1 | 2 | 3 | 4 | 5 | 6 | 7 |
| 11. I can anticipate when a situation will stress me. | 1 | 2 | 3 | 4 | 5 | 6 | 7 |
| 12. I notice possible difficult situations early. | 1 | 2 | 3 | 4 | 5 | 6 | 7 |
| 13. I prepare myself for upcoming challenges. | 1 | 2 | 3 | 4 | 5 | 6 | 7 |

**The Resilience Process Scales (RPS).**

**Emotional**

Please think of different tough **emotional** situations, life events, challenges and obstacles you may have experienced in the past or may experience in the future. This could be *any* situation in which you have felt or may feel anxious, angry, sad, afraid or emotionally stressed.

In these situations…

| In these situations… | Strongly Disagree | | | Neither agree or disagree | | | Strongly Agree |
|---|---|---|---|---|---|---|---|
| 1. I remain positive, even when things seem hopeless. | 1 | 2 | 3 | 4 | 5 | 6 | 7 |
| 2. I make back-up plans for when things might go wrong. | 1 | 2 | 3 | 4 | 5 | 6 | 7 |
| 3. I tend to organise myself well to deal with challenges. | 1 | 2 | 3 | 4 | 5 | 6 | 7 |
| 4. I bounce-back easily after a challenge. | 1 | 2 | 3 | 4 | 5 | 6 | 7 |
| 5. When things get bad, I don't let them get to me. | 1 | 2 | 3 | 4 | 5 | 6 | 7 |
| 6. I can anticipate when help is going to be needed. | 1 | 2 | 3 | 4 | 5 | 6 | 7 |
| 7. I keep a clear head under pressure. | 1 | 2 | 3 | 4 | 5 | 6 | 7 |
| 8. I quickly get over set-backs | 1 | 2 | 3 | 4 | 5 | 6 | 7 |
| 9. I give my best effort no matter the obstacle. | 1 | 2 | 3 | 4 | 5 | 6 | 7 |
| 10. I know how to stop the same things getting to me in the future. | 1 | 2 | 3 | 4 | 5 | 6 | 7 |
| 11. I can anticipate when a situation will stress me. | 1 | 2 | 3 | 4 | 5 | 6 | 7 |
| 12. I notice possible difficult situations early. | 1 | 2 | 3 | 4 | 5 | 6 | 7 |
| 13. I prepare myself for upcoming challenges. | 1 | 2 | 3 | 4 | 5 | 6 | 7 |

Scoring (create a mean for each process).

Anticipate: Q6, Q11, Q12

Minimise: Q2, Q3, Q13

Manage: Q1, Q5, Q9

Mend: Q4, Q8, Q10
