# Supplementary material for: Mapping resilience: Development of the resilience process scales (RPS) and resilience profiles during adversity
Source: PLoS One. 2026 Feb 11;21(2):e0341581. doi: 10.1371/journal.pone.0341581 (PMC12893550; doi:10.1371/journal.pone.0341581)
Supplement: S3 Appendix — Additional details and findings in Study 1. (PDF) [file pone.0341581.s003.pdf]

Study 1: Participant demographics, internal consistency, and interaction effects and differences across resilience processes and domains

Participants demographics

Participants stated their ethnicities as, 1 of them Afro-Caribbean, 2 Asian, 6 mixed, and 172 White, and stated their Nationalities with 176 of them British, 1 Caribbean, 1 Irish, 1 New Zealander, 1 Polish, and 1 Tanzanian.

Internal consistency

Composite reliability coefficient of the anticipation, minimize, manage, and mend subscales were, for the general domain, anticipate: .72, minimize: .78, manage: .76, and mend: .81. For the physical domain, anticipate: .81, minimize: .84, manage: .80, and mend: .81. For the social domain, anticipate: .84, minimize: .86, manage: .80, and mend: .81. For the cognitive domain, anticipate: .81, minimize: .90, manage: .81, and mend: .81. finally, for the emotional domain, anticipate: .76, minimize: .87, manage: .81, and mend: .81.

Interaction effects and differences

Further analysis examined the main differences within the processes and domains to provide relative reference scores and baselines for future studies. This analysis allowed for the exploration and clarification of these domains and processes as distinct from each other – despite relatively high correlations between the processes during the Bayesian analysis. A repeated measures (process × domain) ANOVA revealed a main effect for processes F (2.28, 395.87) = 3.42, $p = .028$, $\eta^2 = .019$, domain F (3.54, 615.48) = 14.24, $p < .001$, $\eta^2 = .076$, and

a processes × domain interaction, F (10.37, 1804.62) = 2.49, p = .014, η^2 = .014. Follow-up tests revealed that within the *processes*, the anticipate subscale was the highest score (M = 4.81), and was significantly higher than mend (M = 4.64; p = .023). Follow-up testing on the domains showed that the physical subscale was highest (M = 4.91), followed by cognitive (M = 4.80), with emotional the lowest (M = 4.64). Physical was significantly higher than general (M = 4.77; p = .039), social (M = 4.62; p < .001), cognitive (p = .012), and emotional (p < .001). Cognitive was significantly higher than social (p = .013) and emotional (p < .001). General was significantly higher than emotional (p < .001). Further, Follow-up tests on the interaction showed that in general domain, anticipate was significantly higher than manage (M_{diff} = 0.22, t = 3.197, d = 0.204) and mend (M_{diff} = 0.29, t = 4.361, d = 0.270). Within the cognitive domain, minimize was significantly higher than manage (M_{diff} = 0.19, t = 2.784, d = 0.164), and within the emotional domain, anticipate was significantly higher than mend (M_{diff} = 0.22, t = 3.226, d = 0.185).
