# Supplementary material for: Mapping resilience: Development of the resilience process scales (RPS) and resilience profiles during adversity
Source: PLoS One. 2026 Feb 11;21(2):e0341581. doi: 10.1371/journal.pone.0341581 (PMC12893550; doi:10.1371/journal.pone.0341581)
Supplement: S4 Appendix — Additional details and findings in Study 2. (PDF) [file pone.0341581.s004.pdf]

Study 2: Participant demographics, interaction effects and differences across resilience processes and domains

Participants demographics

Participants stated their ethnicities as, 3 of them Afro-Caribbean, 47 Asian, 3 mixed, 222 White, and 9 preferred not to say. Their Nationalities were varied, with 13 of them American, 1 Austrian, 1 Bahraini, 1 Belgian, 171 British, 2 Canadian, 36 Chinese, 1 Danish, 1 French, 5 German, 3 Greek, 3 Indian, 1 Iranian, 3 Irish, 6 Italian, 1 Japanese, 1 Lithuanian, 1 Malaysian, 1 New Zealander, 1 Nigerian, 4 Pakistani, 6 Polish, 1 Portuguese, 1 Romanian, 1 Russian, 1 Saudi Arabian, 4 Singaporean, 2 Spanish, 1 Turkish, 1 Vietnamese, and 9 preferred not to say.

Interaction effects and differences

Further analysis examined the main differences within the processes and domains to provide reference scores and baselines for future studies. In addition, it allowed for exploration and clarification of these processes and domains as distinct. A repeated measures (process × domain) ANOVA revealed main effects for process F (2.42, 684.87) = 27.81, $p < .001$, $\eta^2 = .089$, domain F (4, 835.01) = 14.76, $p < .001$, $\eta^2 = .500$, and interaction F (9.12, 2580.66) = 2.54, $p = .007$, $\eta^2 = .009$. Follow-up tests revealed that overall, within the *processes*, the anticipate subscale was the highest score ($M = 5.03$), approaching significance in higher score over minimize ($M = 4.92$; $p = .055$), and significantly higher than manage ($M = 4.75$; $p < .001$), and mend ($M = 4.68$; $p < .001$). Minimize was second highest, significantly higher than manage ($p = .001$) and mend ($p < .001$). Follow-up testing on the domains showed the physical subscale was highest ($M = 5.06$), followed by general ($M = 4.92$), with

emotional the lowest ($M = 4.68$). Physical was significantly higher than general ($p = .022$), social ($M = 4.70$; $p < .001$), cognitive ($M = 4.80$; $p = .012$), and emotional ($p < .001$). General was significantly higher than social ($p = .001$) and emotional ($p = .022$). Cognitive was significantly higher than social ($p = .017$) and emotional ($p = .015$). Further, post-hoc testing on the interaction showed that in the general domain, anticipate was significantly higher than manage ($M_{diff} = 0.23$, $t = 3.61$, $d = 0.216$) and mend ($M_{diff} = 0.25$, $t = 3.92$, $d = 0.237$), and minimize was significantly higher than mend ($M_{diff} = 0.20$, $t = 3.14$, $d = 0.188$). In the physical domain, anticipate was significantly higher than manage ($M_{diff} = 0.56$, $t = 8.79$, $d = 0.509$), and minimize was significantly higher than manage ($M_{diff} = 0.48$, $t = 7.53$, $d = 0.400$). In the social domain, anticipate was significantly higher than manage ($M_{diff} = 0.35$, $t = 5.49$, $d = 0.305$) and mend ($M_{diff} = 0.46$, $t = 7.22$, $d = 0.382$), and minimize was significantly higher than mend ($M_{diff} = 0.29$, $t = 4.55$, $d = 0.223$). In the cognitive domain, anticipate was significantly higher than manage ($M_{diff} = 0.30$, $t = 4.71$, $d = 0.262$) and mend ($M_{diff} = 0.36$, $t = 5.65$, $d = 0.309$), and minimize was significantly higher than manage ($M_{diff} = 0.28$, $t = 4.39$, $d = 0.232$) and mend ($M_{diff} = 0.34$, $t = 5.33$, $d = 0.278$). Lastly in the emotional domain, anticipate was significantly higher than manage ($M_{diff} = 0.32$, $t = 5.02$, $d = 0.269$) and mend ($M_{diff} = 0.46$, $t = 7.22$, $d = 0.371$), and minimize was significantly higher than mend ($M_{diff} = 0.22$, $t = 5.65$, $d = 0.285$).
