# Supplementary material for: Mapping resilience: Development of the resilience process scales (RPS) and resilience profiles during adversity
Source: PLoS One. 2026 Feb 11;21(2):e0341581. doi: 10.1371/journal.pone.0341581 (PMC12893550; doi:10.1371/journal.pone.0341581)
Supplement: S5 Appendix — Examination of hypothesized resilience profiles based on the literature. (PDF) [file pone.0341581.s005.pdf]

Study 3 pilot study: Hypothesised profiles

Low Resilience – Ant Dominant. It seems likely that some individuals may have low resilience across most processes (seeing themselves as having low resilience). This may be particularly prominent during the pandemic, in which resilience levels have been reportedly lower than average [1]. However, an elevated anticipation could be predicted in such an individual. This profile would be consistent with individuals who are sensitive to threats and stress and might over-anticipate adversity, yet do not have the resources to either minimize the impact of the stressor, or to deal with it in the moment [2–4]. This would also result in anticipation becoming a necessity, if they struggle to deal with stressors when they do impact them (particularly being wary of intense stressors). Such individuals would likely experience intense negative emotions in anticipation of events [5].

Moderate Resilience – High Pro-Active. A large portion of the population would likely fit somewhere within the 'average' level of resilience [6, 7]. However, it would be generally expected that processes with higher temporal proximity (e.g., anticipate & minimize) would correlate to a greater degree as they would involve more related abilities in comparison to more distal processes. An individual with higher levels of anticipate and minimize (pro-active components) could reduce how much their more reactive abilities get utilized. This could result in the pro-active components becoming better 'practiced' in comparison to their reactive abilities, potentially leading to further higher pro-active abilities (or perception of) due to more experience using them [8–10].

Moderate Resilience – High Reactive. Following a similar logic to the previously predicted profile, it seems plausible that some individuals who fail to anticipate and minimize the impact of stressors would, by necessity, utilize managing and mending to a greater degree. Individuals would likely fit this profile due to either perceiving these pro-active

Classification: Controlled

components not to be necessary as they manage and mend so effectively, or that these become better practiced out of necessity due to a lack of ability to anticipate and minimize.

High Resilience. Lastly, it seems reasonable to suggest that some individuals would be high across all resilience processes, as we do see many individuals demonstrate high resilience across diverse situations [8, 11]. In the resilience literature, these people are classically referred to as apparently 'invulnerable' individuals and tend to have high self-esteem, self-efficacy, planning and decision-making skills, and supportive social contacts [12, 13]. These consistent protective qualities help these individuals utilize a more challenge mindset when approaching adversity and stressful environments, therefore making them more facilitative to their resilience [9].

Method

Following institutional ethical approval, we recruited a convenience sample of 555 participants (M_{age} = 20.9, *SD* = 8.2; *n* = 263 Male, *n* = 289 Female, *n* = 3 preferred not to say) via social media from the public, secondary schools, and Universities across the UK, and prior to the events of the pandemic.

Measures and procedure

We used the general scale from the Resilience Process Scales (see Studies 1 & 2) to examine the four processes of anticipate, minimize, manage, and mend. Each of the 13 items are measured on a.7-point Likert-type scale (1 = *strongly disagree*, 4 = *neither agree nor disagree*, 7 = *strongly agree*). Participants completed this measure in their own time.

Analysis

We conducted Latent Profile Analysis following the same procedure as Study 3.

Classification: Controlled

Results

The process of LPA resulted in a four-class solution (see Table below). The table presents model fit indices, with the 4-class solution demonstrating lower AIC, BIC, and aBIC in comparison to other models, as well as a good entropy value (.82), and significant LMR LRT and BLRT scores ($p < .05$). Thus, the fit indices provided preliminary evidence that four profiles show a better fit than smaller class solutions (such as the three profiles), with a clearer delineation of profiles within the sample, and an acceptably high group membership within each profile of them. A five-profile solution also had a good fit. However, a combination of less meaningful group memberships (two of the five profiles only represented 7% & 4.9% of the total sample) and slightly lower entropy values in the five-profile solution, coupled with greater model complexity (because of a greater number of profiles), meant that, a four-profile model was used.

Classification: Controlled
