# Supplementary material for: Mapping resilience: Development of the resilience process scales (RPS) and resilience profiles during adversity
Source: PLoS One. 2026 Feb 11;21(2):e0341581. doi: 10.1371/journal.pone.0341581 (PMC12893550; doi:10.1371/journal.pone.0341581)
Supplement: S6 Appendix — Additional participant details, alongside validating and screening preventative behavior, negative affect, and past COVID infection, in addition to screening for extraneous influences. (PDF) [file pone.0341581.s006.pdf]

Study 3: Participant demographics, additional measure validation, and screening

Participant demographics

Participants stated their Nationalities as, 39 of them American, 1 Austrian, 248 British, 1 Croatian, 8 French, 1 Georgian, 1 Greek, 1 Indian, 1 Iranian, 1 Irish, 3 Italian, 1 Malaysian, 1 Norwegian, 1 Polish, 1 Romanian, 1 Spanish, and 1 Sri Lankan.

Preventative behaviors measure validation

The measure for affect was adapted without providing evidence of any validation studies, so we did this ourselves using BSEM (see Study 1 for more details on this approach) and composite reliability. The initial model fit for the preventative behaviors scale was acceptable (PP*p* of .52; CI of -29.29 and 28.25). However, three items had particularly low FLs (<.4), and we deemed less appropriate (e.g., "Used antibiotics to prevent or treat COVID-19" was not deemed a useful preventative behavior). We subsequently removed these items and re-analysed the scale, which resulted in a good model fit (PP*p* of .50; CI of -20.50 and 21.21). All remaining FLs were between .41 to .60, and composite reliability was appropriate at .71.

Negative affect and past COVID infection

The World Health Organisation's recommended COVID-19 survey tool [14] was used to measure affect and past COVID infection. Affect was adapted from Bradley and Lang [15] with COVID-19 as the subject, with seven items such as "COVID-19 to me feels: 1 = *Fear-inducing* to 7 = *Not fear-inducing*" (this item being reverse scored). Past COVID infection was simply asking if participants had been previously infected (measured by *Yes* or *No*),

followed by if it was *mild* or *severe*, and if it was confirmed by a test or not. This was then followed up with if anyone in their immediate social environment *were* or *had been* infected, and if anyone they know had died from it (both being *Yes* or *No* items).

Confirmatory factor analysis, validation, and screening method

The measure for affect was adapted without providing evidence of any validation studies, so we did this ourselves using BSEM (see Study 1 for more details on this approach) and composite reliability. Confirmatory Factor Analysis for the affect scale demonstrated a good model fit (PP*p* of .49; CI of -20.65 and 20.57) and Factor Loadings (FL) between .49 to .64, with a composite reliability score of .72 for all items.

We then explored the extent to which affect and whether the participant had been infected with COVID-19 influenced profile membership using the R3STEP command [16] as an addition to LPA. We also conducted correlational analysis between each of these measures and the separate resilience processes, to examine if these variables could have influenced resilience levels and profile membership.

Screening for extraneous influence results

Affect was negatively correlated with previous COVID-19 infection ($r = -.26$; $p < .001$), and positively correlated with anticipate ($r = .16$; $p = .002$) and minimize ($r = .20$; $p < .001$). Further, infection and minimize were negatively correlated ($r = -.15$; $p = .004$).

R3step LPA gave odds ratio tests demonstrating that a high negative affect would make an individual more likely to be in Profile 2 than Profile 3 ($OR = .71$; $p = .006$), suggesting that individuals reporting more negative affect tended to have a lower resilience profile. People who had had COVID-19 before were more likely be in Profile 1 than Profile 2 ($OR = .26$; $p < .001$), Profile 3 ($OR = .35$; $p = .017$), or Profile 4 ($OR = .12$; $p < .001$. In

addition, they were more likely to be in Profile 2 than Profile 4 (*OR* = .33; *p* = .002). Thus, individuals reporting previous infections were more likely to be in a lower resilience profile than high resilience.
