# Supplementary material for: Mapping resilience: Development of the resilience process scales (RPS) and resilience profiles during adversity
Source: PLoS One. 2026 Feb 11;21(2):e0341581. doi: 10.1371/journal.pone.0341581 (PMC12893550; doi:10.1371/journal.pone.0341581)
Supplement: S7 Appendix — Findings for causal outcomes of resilience profiles for depression, well-being, risk-taking, and impulsiveness. (PDF) [file pone.0341581.s007.pdf]

Study 3: Causal outcomes of resilience profiles

Depression

The profiles still demonstrated significant differences in depression ($\chi2 = 11.73$, $p = .008$). Profile 4 ($M = 3.05$) displayed the lowest score, and Profile 1 ($M = 4.42$) the highest once again. Those in Profile 1 were significantly higher in depression than those in Profile 2 ($M_{diff} = 0.90$, $p = .031$) and 4 ($M_{diff} = 1.38$, $p = .001$), and those in Profile 3 were significantly higher than those in Profile 4 ($M_{diff} = 0.85$, $p = .010$).

Well-being

The profiles showed significant differences in well-being ($\chi2 = 36.30$, $p < .001$). Profile 4 ($M = 94.57$) displayed the highest score, and Profile 3 ($M = 65.25$) the lowest. Those in Profile 4 were significantly higher than those in all other profiles (vs. Profile 1: $M_{diff} = 26.49$, $p = .011$; Profile 2: $M_{diff} = 22.49$, $p = .011$; Profile 3: $M_{diff} = 29.32$, $p = .011$), those in Profile 2 were also significantly higher than those in Profile 3 ($M_{diff} = 6.84$, $p = .011$).

Risk-taking

The profiles again showed significant differences in risk-taking, with a similar pattern across the four profiles ($\chi2 = 12.98$, $p = .005$). Profile 4 ($M = 3.44$) displayed the highest score, and Profile 1 ($M = 2.69$) the lowest. Members of Profile 4 were significantly higher in risk taking than those in Profile 1 ($M_{diff} = 0.75$, $p = .007$), those in Profile 2 were significantly lower than those in Profile 3 ($M_{diff} = 0.15$, $p = .047$) and higher than those in Profile 1 ($M_{diff} = 0.53$, $p = .010$), and those in Profile 3 were significantly higher than those in Profile 1 ($M_{diff} = 0.68$, $p = .001$).

Classification: Controlled

Impulsiveness

The profiles still showed significant differences in impulsiveness, with a similar pattern across the four profiles ($\chi2 = 10.18$, $p = .017$). Profile 2 ($M = 19.57$) displayed the highest score, and Profile 4 ($M = 15.77$) the lowest. Those in Profile 2 were significantly higher than those in Profile 3 ($M_{\text{diff}} = 0.95$, $p = .030$) and Profile 4 ($M_{\text{diff}} = 3.80$, $p = .014$).

Classification: Controlled
