# Supplementary material for: Mapping resilience: Development of the resilience process scales (RPS) and resilience profiles during adversity
Source: PLoS One. 2026 Feb 11;21(2):e0341581. doi: 10.1371/journal.pone.0341581 (PMC12893550; doi:10.1371/journal.pone.0341581)
Supplement: S8 Appendix — Additional references cited throughout supporting information materials. (PDF) [file pone.0341581.s008.pdf]

Supplementary references

1. Killgore WDS, Taylor EC, Cloonan SA, Dailey NS. Psychological resilience during the COVID-19 lockdown. Psychiatry Res. 2020;291:113216. doi:10.1016/j.psychres.2020.113216.

2. Dickson KS, Ciesla JA, Reilly LC. Rumination, worry, cognitive avoidance, and behavioural avoidance: examination of temporal effects. Behav Ther. 2012;43(3):629–40. doi:10.1016/j.beth.2011.11.002.

3. Gross JJ. Emotional regulation: affective, cognitive, and social consequences. Psychophysiology. 2002;39(3):281–91. doi:10.1017/S0048577201393198.

4. Schneider TR, Lyons JB, Khazon S. Emotional intelligence and resilience. Pers Individ Dif. 2013;55(8):909–14. doi:10.1016/j.paid.2013.07.460.

5. Van Boven L, Ashworth L. Looking forward, looking back: anticipation is more evocative than retrospection. J Exp Psychol Gen. 2007;136(2):289–300. doi:10.1037/0096-3445.136.2.289.

6. Cigna. Cigna Resilience Index (2020 U.S. Report); 2020. Available from: https://cignaresilience.com/

7. Kocalevent RD, Zenger M, Heinen I, Dwinger S, Decker O, Brahler E. Resilience in the general population: standardization of the Resilience Scale (RS-11). PLoS One. 2015;10(11):e0140322. doi:10.1371/journal.pone.0140322.

8. Barrett EC, Martin P. Extreme: why some people thrive at the limits. Oxford University Press; 2014.

9. Fletcher D, Sarkar M. Mental fortitude training: An evidence-based approach to developing psychological resilience for sustained success. Journal of Sport Psychology in Action. 2016;7(3):135–157. doi:10.1080/21520704.2016.1255496

10. Meichenbaum D, Novaco R. Stress inoculation: a preventative approach. Issues Ment Health Nurs. 1985;7(1–4):419–35. doi:10.3109/01612848509009464.

11. Pangallo A., Zibaras L, Lewis R, Flaxman P. Resilience through the lens of interactionism: a systematic review. Psychological assessment. 2015;27(1):1–20. https://doi.org/10.1037/pas0000024

12. Fleming J, Ledogar RJ. Resilience, an evolving concept: a review of literature relevant to Aboriginal research. Pimatisiwin. 2008;6(2):7–23.

13. Olsson CA, Bond L, Burns JM, Vella-Brodrick DA, Sawyer SM. Adolescent resilience: a concept analysis. J Adolesc. 2003;26(1):1–11. doi:10.1016/S0140-1971(02)00118-5.

14. World Health Organization. Survey tool and guidance; 2020. Available from: https://www.euro.who.int/__data/assets/pdf_file/0007/436705/COVID-19-survey-tool-and-guidance.pdf

15. Bradley MM, Lang PJ. Measuring emotion: The self-assessment manikin and the semantic differential. Journal of Behavior Therapy and Experimental Psychiatry. 1994;25(1):49–59. 10.1016/0005-7916(94)90063-9

16. Asparouhov T, Muthén B. Auxiliary variables in mixture modeling: three-step approaches using Mplus. Struct Equ Model. 2014;21(3):329–41. doi:10.1080/10705511.2014.915181.
