# Supplementary material for: Mapping resilience: Development of the resilience process scales (RPS) and resilience profiles during adversity
Source: PLoS One. 2026 Feb 11;21(2):e0341581. doi: 10.1371/journal.pone.0341581 (PMC12893550; doi:10.1371/journal.pone.0341581)
Supplement: S1 Table — Table showing Study 1 correlations across resilience processes and domains. (PDF) [file pone.0341581.s009.pdf]

Study 1 correlations

S1 Table. Correlations across resilience processes and domains.

**General**	Anticipate	Minimize	Manage	Mend
Anticipate	1			
Minimize	0.32*	1		
Manage	0.31*	0.39*	1	
Mend	0.46**	0.40*	0.69***	1
**Physical**				
Anticipate	1			
Minimize	0.51**	1		
Manage	0.68***	0.57***	1	
Mend	0.57***	0.67***	0.62***	1
**Social**				
Anticipate	1			
Minimize	0.62***	1		
Manage	0.71***	0.58***	1	
Mend	0.54***	0.61***	0.59***	1
**Cognitive**				
Anticipate	1			
Minimize	0.57***	1		
Manage	0.70***	0.58***	1	
Mend	0.63***	0.64***	0.69***	1
**Emotional**				

Classification: Controlled

Anticipate	1			
Minimize	0.63***	1		
Manage	0.67***	0.63***	1	
Mend	0.68***	0.76***	0.70***	1

Note. p < .05*; p < .01**; p < .001***

Classification: Controlled
