# Supplementary material for: Mapping resilience: Development of the resilience process scales (RPS) and resilience profiles during adversity
Source: PLoS One. 2026 Feb 11;21(2):e0341581. doi: 10.1371/journal.pone.0341581 (PMC12893550; doi:10.1371/journal.pone.0341581)
Supplement: S2 Table — Table showing Study 2 correlations across resilience processes and domains. (PDF) [file pone.0341581.s010.pdf]

Study 2 correlations

S2 Table. Correlations across resilience processes and domains.

**General**	Anticipate	Minimize	Manage	Mend
Anticipate	1			
Minimize	0.44**	1		
Manage	0.40**	0.48***	1	
Mend	0.46**	0.51***	0.77***	1
**Physical**				
Anticipate	1			
Minimize	0.60***	1		
Manage	0.52***	0.68***	1	
Mend	0.57***	0.69***	0.83***	1
**Social**				
Anticipate	1			
Minimize	0.59***	1		
Manage	0.49**	0.59***	1	
Mend	0.54***	0.58***	0.73***	1
**Cognitive**				
Anticipate	1			
Minimize	0.58***	1		
Manage	0.49***	0.62***	1	
Mend	0.47**	0.65***	0.79***	1
**Emotional**				

Classification: Controlled

Anticipate	1			
Minimize	0.54***	1		
Manage	0.51***	0.59***	1	
Mend	0.52***	0.58***	0.79***	1

Note. $p < .05$*; $p < .01$**; $p < .001$***

Classification: Controlled
