# Supplementary material for: Mapping resilience: Development of the resilience process scales (RPS) and resilience profiles during adversity
Source: PLoS One. 2026 Feb 11;21(2):e0341581. doi: 10.1371/journal.pone.0341581 (PMC12893550; doi:10.1371/journal.pone.0341581)
Supplement: S3 Table — Fit indices comparing profile solutions. (PDF) [file pone.0341581.s011.pdf]

Study 3 pilot study: Fit indices

S3 Table. Fit indices comparing profile solutions.

LPA Outputs	AIC	BIC	Adj BIC	Entropy	LMR LRT	BLRT
1 Class	6522.35	6556.9	6531.5	N/A	N/A	N/A
2 Class	6066.93	6123.07	6081.81	0.758	<.001	<.001
3 Class	5886.08	59.63.82	5906.68	0.763	0.7897	<.001
4 Class	5749.19	5848.52	5775.51	0.818	0.02	<.001
5 Class	5699.57	5820.5	5731.62	0.796	0.0191	<.001

Classification: Controlled
