# Supplementary material for: Mapping resilience: Development of the resilience process scales (RPS) and resilience profiles during adversity
Source: PLoS One. 2026 Feb 11;21(2):e0341581. doi: 10.1371/journal.pone.0341581 (PMC12893550; doi:10.1371/journal.pone.0341581)
Supplement: S1 Fig — Standardized profiles of resilience for pilot study. (PDF) [file pone.0341581.s012.pdf]

Study 3 pilot study: Standardized profiles

S1 Fig. Standardized profiles of resilience for pilot study.

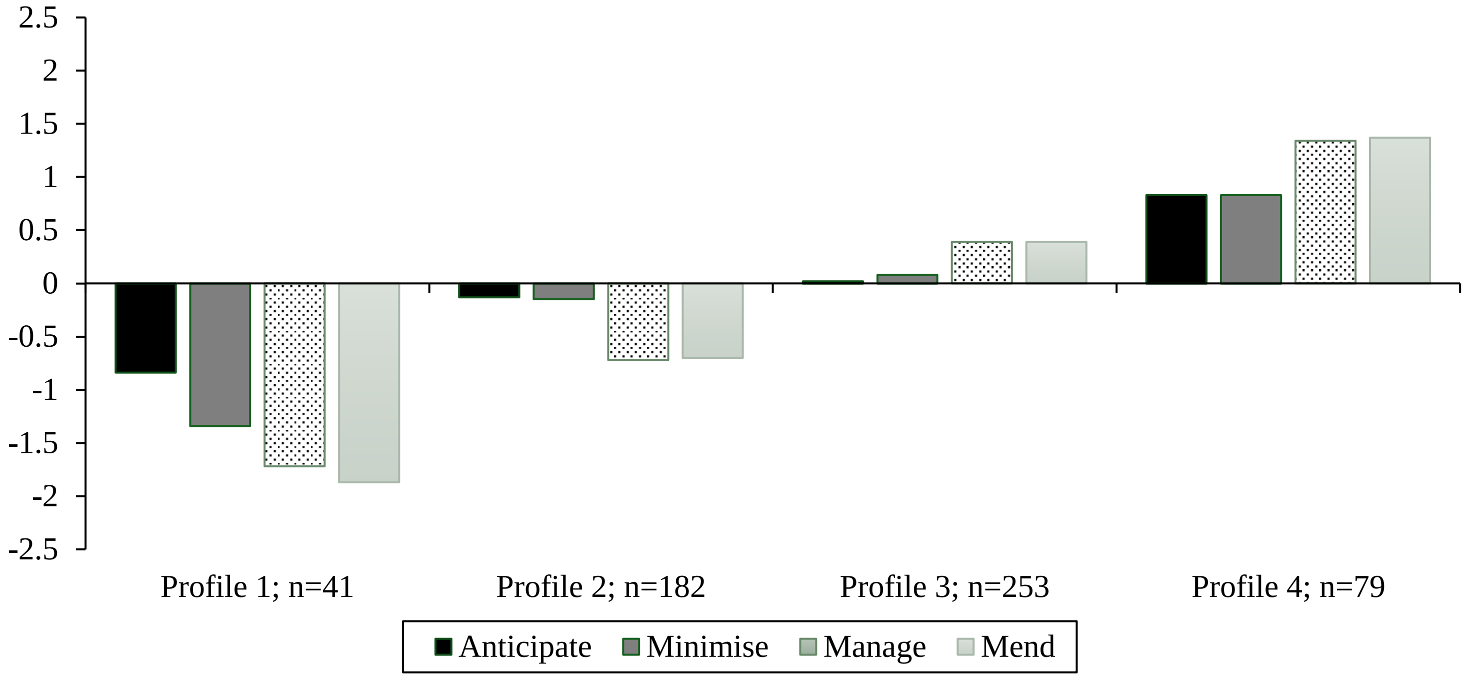

Pilot study standardized profiles (*n* = 555) by Profile 1 (Low Resilience – High Anticipate), Profile 2 (Low Resilience – High Pro-Active), Profile 3 (Moderate Resilience), and Profile 4 (High Resilience – High Reactive).

Classification: Controlled
