# Supplementary material for: Mapping resilience: Development of the resilience process scales (RPS) and resilience profiles during adversity
Source: PLoS One. 2026 Feb 11;21(2):e0341581. doi: 10.1371/journal.pone.0341581 (PMC12893550; doi:10.1371/journal.pone.0341581)
Supplement: Attachment — Submitted filename: Response to Reviewers.docx [file pone.0341581.s014.docx]

**Response to Reviewers**

We would like to thank the associate editor and all reviewers for their detailed and helpful comments. We have made a number of changes in light of these comments and believe that the manuscript is now much stronger as a result. We provide details below as to how we have addressed the comments (note that when referring to where to find these changes, we are referring to the Revised Manuscript with Track Changes document). Please note that we did not track changes with the amended reference list as we felt this made the document unnecessarily long and unwieldy.

Reviewer Comments and Responses

We would like to thank you for the detailed review and feedback you have provided. We have since made a number of changes to the manuscript and feel that the revision is much better as a result.

Reviewer #1

| 1. Strengthen the reasoning for treating "anticipate" and "minimize" as separate proactive processes. Clearly contrast this with Chen et al.'s (2010) combined "anticipate" factor and Alliger et al.'s (2009) framework. Present empirical and theoretical evidence (like appraisal theory) early in the Introduction. | Response: We have added further detail and evidence to this section in the Introduction (pages 2 & 3). It is worth noting our intention is to highlight the proposed model adds to and extends the work or Chen et al. and Alliger et al. rather than contrasting it. |
| --- | --- |
| 2. Clarify the "cognitive resilience" area. While it's claimed to be novel, explain how it differs from "emotional" resilience. Provide stronger theoretical support (beyond job-related examples) and describe cognitive processes (like executive function under stress) apart from emotional regulation. | Response: We have added more detail and evidence to this section in the Introduction, alongside detail on its distinction from emotional resilience (pages 4 & 5) to make this point clearer. |
| 3. Detail how items were reduced: The manuscript notes items were cut from 94 to 65 based on wording and similarity, then to 20 through expert ratings, and finally to 13 using BSEM. Provide: The criteria used for expert ratings (such as inter-rater agreement statistics). A clear justification (beyond factor loadings) for keeping items with correlated residuals that exceed the usual thresholds. A table listing all initial items along with reasons for exclusion in the supplementary materials. | Response: In our attempt to try and be as transparent as possible regarding measurement development, we realise we may have actually made the reporting of our process less clear. We apologise for this and appreciate you raising your points, which has given us an opportunity to reflect on what we originally reported. We now have re-written this section (pages 6 & 7) to be more accurate and easier to follow.  We began the process by examining items from existing resilience scales, and found 94 in total. Using these items as a foundation, we then completed a process of item modification or creation to leave us with 65 items that we felt could map onto each of our resilience processes and to each domain.  Following an initial review of content validity – where we looked at inter-rater reliability regarding how effective we felt each item was to its process and domain, we then presented our potential items to a panel of experts in psychology with over 100 combined years of experience in research and practice as well as two expedition leaders. The conclusion from this presentation was that it would be better to use the same items across domains (alongside having a general domain), as having different items across different domains would prevent researchers being able to compare individual responses across each domain.  As such we re-worked our items from these discussions and the inter-rater reliability scores down to 20 items that we felt clearly aligned to each mechanism and would be appropriate for each domain, with the idea that domain specific responses could be provided by using a vignette. In order to keep the manuscript focused and concise we haven’t provided all of this information (we are also mindful of comments from the other reviewers regarding manuscript length); rather we have given a summary of the initial work and then provided the original 20 item measure in the supplementary information (S1 Appendix). Given that our focus changed across the process we do not believe it would be helpful to include the initial 65 items that we developed as these are not relevant. We hope you find our approach appropriate. |
| 4. Explain the BSEM method: Briefly clarify why BSEM was more suitable than frequentist SEM (for example, for managing small cross-loadings) for PLOS ONE's wider readership. Clearly outline key prior specifications. | Response: We have added more detail regarding the benefits of the BSEM approach over the frequentist approach to CFA and SEM on page 10. |
| 5. Include the full vignettes for each domain in the main text or a well-referenced supplement (S1 Appendix). The wording is important for understanding domain scores. | Response: We have now added the full vignette from the general domain, alongside clear reference to where they can find the other domain-specific vignettes (pages 8 & 9) in the supplements (S2 Appendix). |
| 6. Acknowledge that the sample limitations in Studies 1 and 2 (expedition participants, UK students/community) affect generalizability. Discuss possible biases, such as the expedition group potentially having higher baseline resilience. | Response: Thank you for these observations, we agree with them. We have edited the discussion (see page 50) to reflect the points raised. |
| 7. Reconcile pilot and main profiles: The main study identified a "Low Resilience – Low Anticipate" profile (36.5%) that was not predicted in the pilot phase. Discuss possible reasons, such as the pandemic context affecting anticipation abilities or differences in samples. | Response: We agree and have added some further discussion of this in the Resilience profiles subsection of the General Discussion (pages 43-47). |
| 8. Consider Profile 4 (High Resilience) size: With just 5.5% of the sample, the statistical power for comparisons involving this profile is low (evident in anxiety SE=1.03). Be cautious in interpreting its "superior" outcomes. Consider merging it with Profile 3 if justifiable or clearly state the limitations in power. | Response: We appreciate your point and have emphasized the limitation in statistical power and caution therefore needed in the Results section of Study 3 (pages 34 & 35), and reminding the reader of this in the General discussion on resilience profiles (pages 43 & 44). |
| 9. The instability in LTA transitions (for example, High Resilience to Lower Resilience) needs a more thorough discussion. Connect this to the proposed state-like aspects of resilience and potential pandemic impacts, like prolonged stress draining resources. Did specific events, such as a COVID infection, relate to these transitions? | Response: We have added a bit more detail to the Resilience profiles section of the General discussion on this (pages 43-46) to give a stronger consideration to the resilience transitions and their implications. This would certainly be an interesting direction for future research to examine further. Though it wasn’t a focus of these studies, we do briefly examine some extraneous variables such as Affect and COVID infection (Appendix S6). |
| 10. Contextualize when data was collected: The data was gathered between Feb and July 2021, during the easing of UK lockdowns and the rollout of vaccines. Note how this specific time, characterized by shifting restrictions and new hope, may have affected perceptions of resilience and outcomes compared to the initial waves of the pandemic. | Response: This is now briefly mentioned as a limitation in the General discussion (pages 44, 49, & 50). |
| 11. Clarify what "preventative behaviors" mean: The factor analysis is in S5 Appendix. Briefly report significant loadings for the final items in the main text for transparency. How were "Not Applicable" responses handled? To control for pandemic impacts, consider adding a brief measure of objective or subjective pandemic hardship (like job loss or the severity of illness) as a covariate in the profile-outcome analyses to isolate resilience effects. | Response: We have added more detail in the Study 3 Measures section around preventative behaviours (pages 29). Regarding the points about possible covariates, we take your perspective on board, as we agree that it would be good to ensure that any effects obtained are related to resilience, and not something else. However, to our knowledge LPA and LTA do not allow the inclusion of covariates (in the same way that ANCOVA or regression analyses do). As such, any “covariates” included into an LPA would actually be included in each profile as a substantive variable. Given that we wanted to focus solely on resilience profiles, including variables such as pandemic hardship within our resilience profiles would be difficult as it would make the profiles difficult to interpret. In order to ensure that other variables were not influencing our findings in any way, we did screen the sample with regards to the influence of possible extraneous variables – in which one could examine if a variable (e.g., COVID-19 infection) could influence profile membership in LPA (Appendix S6) in which we had some interesting findings, but was not the main focus of these studies. We do briefly discuss how exploring antecedents of resilience would be an interesting future direction (page 49). |
| 12. Trait vs. State: The LTA shows both stability and change. Reframe the discussion to better incorporate this, avoiding too much reliance on either trait or state views. Emphasize how profiles may reflect current resource use. | Response: We have changed/added to this section in the General discussion to better reflect the state and trait components of resilience (pages 44-47). |
| 13. Applied Recommendations: Make the recommendations for practical use clearer. How can practitioners address specific processes (like developing "minimizing" strategies through contingency planning)? Link these to existing interventions if possible. | Response: We have added a significant amount to the Applied implications subsection of the General discussion on this topic (pages 46-49), giving clearer recommendations and consideration to the implications for practitioners and interventions. |
| 14. Cognitive Domain Validation: Emphasize that this is just a beginning proposal. Explicitly state that further research is needed to validate it against cognitive performance under stress (for example, working memory tasks). | Response: We appreciate this point, we have emphasized this in the domains and limitations sections of the general discussion (pages 42 & 48). |
| 15. Abstract: State the final sample size for the main analysis in Study 3 (N=400). Specify the key pandemic outcomes where profiles showed significant differences (for example, "Profile 1 reported higher anxiety and depression"). | Response: We have added sample sizes to the abstract, alongside more detail on profile differences. |
| 16. Tables and Figures: For Tables 1 and 2, formatting appears corrupted in the provided text. Ensure tables are clear and legends fully explain abbreviations (like PPP, BSEM, CI) in the final submission. Report factor loadings with standard errors and credibility intervals clearly. For Figures 1-4, make sure profiles are clearly labeled (including N per profile) and processes (Anticipate, Minimize, etc.) are distinct. Include details in the main text if possible. | Response: We have added more detail as you suggest (page 12). The formatting seems to be an issue with the conversion of the format onto the PLOS ONE portal – hopefully this won’t reflect in the finalised paper. We left the note explaining abbreviations at the end of Table 2 for ease – given that this table is also quite long.  We included profile sample distribution more clearly (pages 33, 34, & 39) – this also revealed to us an error on the sample numbers and Fig 1 which we have corrected so thank you for this observation. |
| 17. Measurement: Report internal consistency (Cronbach’s α/ω) for all RPS subscales (Anticipate, Minimize, Manage, Mend) per domain in Studies 1 and 2, not just a range. Mention the response scale for the RPS (1-7 Likert) in the Method section. Clarify if the PHQ-4 was scored as PHQ-2 (depression) and GAD-2 (anxiety) subscales, as implied. | Response: We appreciate the point about transparency and have added all the composite reliability coefficient scores we did in the Appendices (S3) to not detract from the flow of the main paper too much.  The response scale for the RPS is mentioned in the Method (pages 7 & 8), but we have now also mentioned in the added vignette for further clarity and emphasis (pages 8 & 9).  We have also added the other clarifications (page 28) for transparency. |

Reviewer #2

Thank you for the helpful feedback, we hope the extra detail and information meets your expectation to improve the paper.

| 1. While the psychometric and profile analyses are appropriate, the reporting of sampling procedures, attrition, and demographic representation could be strengthened to allow clearer evaluation of generalizability. More detail on how participants were recruited across studies, and how missing data were handled, would improve transparency. | Response: We appreciate your point and have added more detail that we have for the study participants regarding demographics to be as transparent as we can in the Appendices (S3, S4, and S6) so as not to disrupt the flow of the paper too much. |
| --- | --- |
| 2. The exploratory and confirmatory factor analyses are reported appropriately, but clearer justification for the specific factor retention criteria and profile model selection would be helpful. For example, why was the four-profile solution favored over alternatives? Please expand on fit indices and theoretical rationale. In the latent transition analyses, additional detail on model fit, classification accuracy, and transition probabilities would improve rigor. | Response: We have added as much detail as we can regarding the approach and process taken (pages 30-33). |
| 3. The conclusions are generally supported by the data, but some claims (for example, implications for clinical interventions or broad generalizations about resilience processes) should be framed more cautiously. Given the reliance on self-report and the COVID-specific context, the authors should take care not to overstate the generalizability of the findings. | Response: We appreciate your point and have added additional points on the generalizability and validity of the study’s findings throughout the discussion and amongst additions we have added (pages 41-50). |
| 4. PLOS ONE requires full availability of the underlying data. At present, it is unclear whether item-level or raw data sufficient to reproduce analyses are accessible. Please ensure that all relevant data and code are made available via a public repository, or provide a clear justification if restrictions apply. | Response: Thank you for bringing this error to our attention. We have now uploaded the data we have (item-level for studies 1 & 2, subfactor level for study 3) to a data repository: <https://osf.io/mtegk> |
| 5. The manuscript is written in clear, standard English and is intelligible. However, some sections (particularly in the Results and Discussion) could be streamlined to improve readability. A careful edit for conciseness would strengthen the presentation. | Response: We have done our best to try to streamline as much of the writing as we can (e.g. page 46), although we should highlight we have added additional detail and information to meet the requests of the other reviewers. We hope you find our approach appropriate. |

Reviewer #3

We would like to thank you for the helpful and detailed review. We have made additions based on your comments that we hope improves our paper. In particular, the resilience framework offered by Farchi & Peled-Avram (2025) was also useful and helpful to consider.

| 1. Differentiation between potential and available coping resources. The model implicitly relies on coping resources, particularly in the anticipatory and minimizing stages. However, the manuscript does not fully distinguish between potential resources (those that are theoretically present or possible) and available resources (those accessible and ready for use in real time). Clarifying this distinction, which has been emphasized in recent resilience frameworks (e.g., Farchi & Peled-Avram, 2025, The ART of Resilience), could make the model more precise and practically relevant. | Response: Thank you for the interesting point and for highlighting this useful work. We have now included some more detail considering it in the Introduction (pages 2 & 3) and General discussion (pages 44, 47, & 49) to bring some of this into the studies. |
| --- | --- |
| 2. Guidance for developing the four resilience processes. While the manuscript convincingly identifies four key domains of resilience, it remains less clear how these processes might be cultivated or strengthened. Including preliminary considerations on pathways for development would broaden the paper’s value for interventions, training, and prevention programs. | Response: We have added a significant amount to the Applied implications subsection (pages 46-50) on this topic and how our model could be applied. However, we should highlight that the focus of our manuscript was not on applied issues directly (rather we wanted to present our model and test its utility), as such we note that the applied issues we cover would be worth exploring fully in a future study. |
| 3. Consideration of the time dimension. The four processes (anticipate → minimize → manage → mend) are presented as a logical sequence, yet the manuscript does not explicitly situate them within different temporal phases of adversity, such as the acute stage, prolonged coping, and post-event recovery. Addressing the time dimension could enrich the model and clarify how these processes function across varying contexts. | Response: We agree this an interesting point and direction, and though not a focus in the current studies, could represent a direction for future research. Nevertheless, we give some consideration to this in the General discussion (page 41). |
| 4. Application to intervention, prevention, and rehabilitation. Although the discussion notes that resilience profiles may guide future interventions, the practical implications remain underdeveloped. Extending the discussion to illustrate how the model might inform strategies for acute intervention, preventive measures, or long-term rehabilitation would significantly strengthen the manuscript’s applied relevance. | Response: We appreciate this point and have added more detail to potential applications to the General discussion (46-50). |
